# Tissue characterization with native T1 mapping in suspected coronary microvascular dysfunction and no obstructive coronary artery disease: results from the NHLBI-sponsored WISE study

**DOI:** 10.1186/1532-429X-18-S1-O43

**Published:** 2016-01-27

**Authors:** Jaime L Shaw, Michael D Nelson, Janet Wei, Puja K Mehta, Louise E Thomson, Daniel S Berman, Debiao Li, C Noel Bairey Merz, Behzad Sharif

**Affiliations:** 1 grid.50956.3f0000000121529905Biomedical Imaging Research Institute, Cedars-Sinai Medical Center, Los Angeles, CA USA; 2 grid.19006.3e0000000096326718Bioengineering, UCLA, Los Angeles, CA USA; 3 grid.50956.3f0000000121529905Barbra Streisand Women's Heart Center, Cedars-Sinai Heart Institute, Los Angeles, CA USA

## Background

About half of all women who present with symptoms of ischemic heart disease have no obstructive coronary artery disease. These women are often deemed "low-risk" patients according to conventional cardiology wisdom. The Women's Ischemia Syndrome Evaluation (WISE) studies have demonstrated suspected coronary microvascular dysfunction (CMD) in women with persistent angina, evidence of ischemia by stress test and no obstructive coronary artery disease (CAD), defined as >50% stenosis. Women with suspected CMD also carry a higher risk of major adverse cardiac events including heart failure (HF). It is also known that diffuse myocardial fibrosis and reduced diastolic function are features in HF. The physiological mechanism behind increased HF in suspected CMD is not known. We assessed the hypothesis that function would be reduced along with previously shown elevated native myocardial T1 in suspected CMD.

## Methods

We evaluated the presence of diffuse fibrosis in women with suspected CMD and healthy controls using native T1 mapping at 1.5T (Siemens Avanto) with a 5(3)3 MOLLI sequence in a single mid-ventricular slice. Functional imaging included short-axis cine images analyzed with feature-tracking software to determine strain and strain rate (cvi42, Circle Cardiovascular Inc.). Suspected CMD subjects had persistent symptoms of angina, evidence of ischemia on stress testing, and no obstructive CAD. Healthy controls had no history of cardiovascular disease and a normal exercise treadmill test.

## Results

Reference control women (n = 11) and women with suspected CMD (n = 28) were well matched in age (50.5 ± 10.4 vs 53.1 ± 12.3, p = 0.53), body mass index (25.9 ± 4.5 vs 25.1 ± 5.3, p = 0.64), and preserved left ventricular ejection fraction (64.5 ± 2.8 vs 61.9 ± 8.4, p = 0.33). We found elevated native T1 values in women with suspected CMD compared to control women (1034.4 ± 32.4 vs 1003.2 ± 19.3 ms, p < 0.01). In subjects with suspected CMD no association was observed between native T1 and age, body mass index, hypertension, Myocardial Perfusion Reserve Index (MPRI, a non-invasive measure of coronary flow reserve), or presence of late gadolinium enhancement. A negative association between native T1 and left ventricular ejection fraction was demonstrated (r = -0.423, p = 0.03). Diastolic circumferential strain rate was impaired in women with suspected CMD compared to controls with a trend towards significance (106.1 ± 26 vs 126.2 ± 37%/s, p = 0.068), consistent with and expanding previous work with tissue tagging in women with suspected CMD.

## Conclusions

Among women with suspected CMD, native myocardial T1 is elevated compared to controls consistent with diffuse fibrosis. The trend towards impaired diastolic circumferential strain rate along with significantly elevated native T1 indicate a possible link of fibrosis and reduced diastolic function in subjects with CMD. Our results further expand on previous work in CMD and may help to elucidate the potential underlying mechanism leading to HF and other adverse events in CMD.

**Figure 1:**
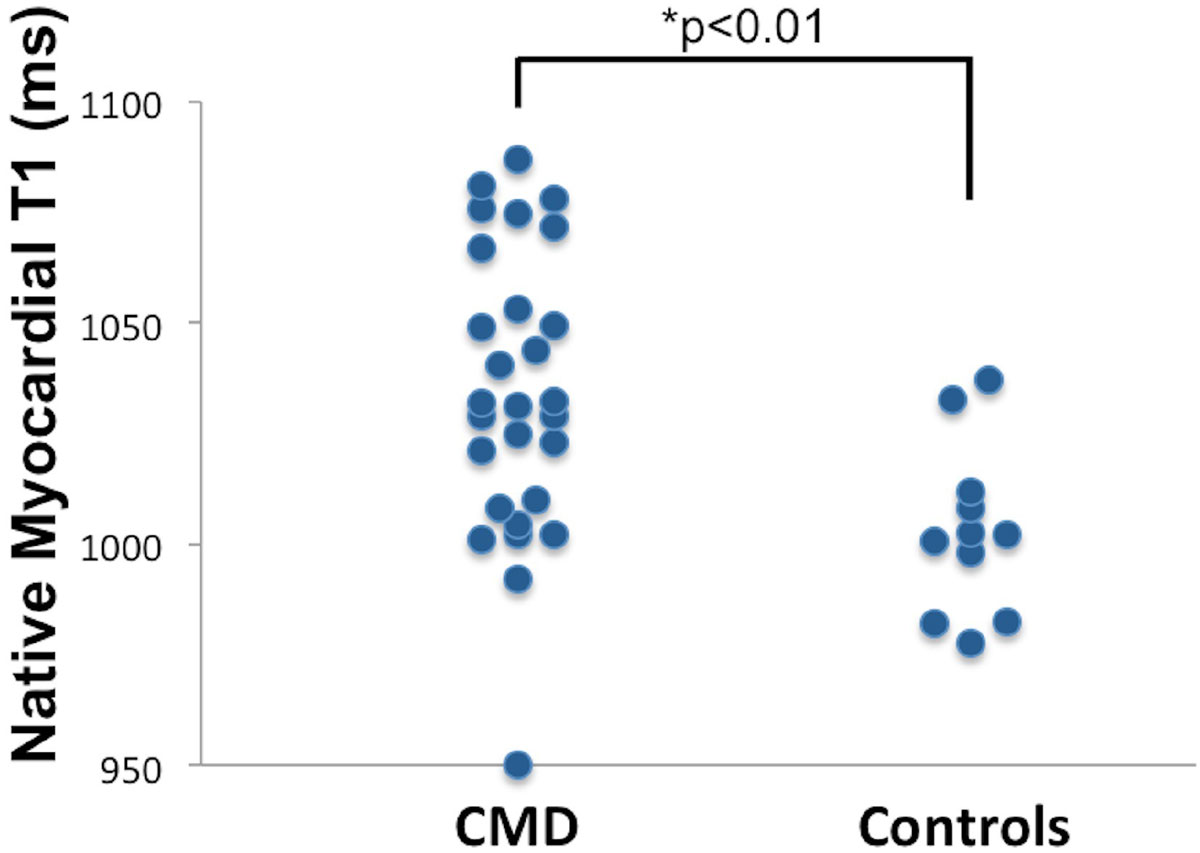
**Native myocardial T1 in subjects with suspected CMD versus healthy controls (1034.4 ± 32.4 vs 1003.2 ± 19.3 ms, p < 0.01)**.

